# Comparative Analysis of the Testis and Ovary Transcriptomes in Zebrafish by Combining Experimental and Computational Tools

**DOI:** 10.1002/cfg.418

**Published:** 2004-07

**Authors:** Yang Li, Jer Ming Chia, Richard Bartfai, Alan Christoffels, Gen Hua Yue, Ke Ding, Mei Yin Ho, James A. Hill, Elia Stupka, Laszlo Orban

**Affiliations:** 1 Reproductive Genomics Group Temasek Lifesciences Laboratory Singapore; 2 Genome Institute of Singapore Singapore; 3 Institute of Molecular and Cell Biology Singapore; 4 Department of Biological Sciences National University of Singapore Singapore; 5 Computational Biology Temasek Lifesciences Laboratory Singapore

## Abstract

Studies on the zebrafish model have contributed to our understanding of several important developmental processes, especially those that can be easily studied in the embryo. However, our knowledge on late events such as gonad differentiation in the zebrafish is still limited. Here we provide an analysis on the gene sets expressed in the adult zebrafish testis and ovary in an attempt to identify genes with potential role in (zebra)fish gonad development and function. We produced 10 533 expressed sequence tags (ESTs) from zebrafish testis or ovary and downloaded an additional 23 642 gonad-derived sequences from the zebrafish EST database. We clustered these sequences together with over 13 000 kidney-derived zebrafish ESTs to study partial transcriptomes for these three organs. We searched for genes with gonad-specific expression by screening macroarrays containing at least 2600 unique cDNA inserts with testis-, ovary- and kidney-derived cDNA probes. Clones hybridizing to only one of the two gonad probes were selected, and subsequently screened with computational tools to identify 72 genes with potentially testis-specific and 97 genes with potentially ovary-specific expression, respectively. PCR-amplification confirmed gonad-specificity for 21 of the 45 clones tested (all without known function). Our study, which involves over 47 000 EST sequences and specialized cDNA arrays, is the first analysis of adult organ transcriptomes of zebrafish at such a scale. The study of genes expressed in adult zebrafish testis and ovary will provide useful information on regulation of gene expression in teleost gonads and might also contribute to our understanding of the development and differentiation of reproductive organs in vertebrates.


**Comparative and Functional Genomics**
*Comp Funct Genom* 2004; **5**: 403–418.
Published online in Wiley InterScience (www.interscience.wiley.com). **DOI:** 10.1002/cfg.418

**Research Article**

### Comparative analysis of the testis and ovary transcriptomes in zebrafish by combining experimental and computational tools

Yang Li[1 #], Jer Ming Chia[2,3#], Richard Bartfai[1,4], Alan Christoffels[3], Gen Hua Yue[1], Ke Ding[1], Mei Yin Ho[1], James A. Hill[1], Elia Stupka[5*] and Laszlo Orban[1,4*]

[1] *Reproductive Genomics Group, Temasek Lifesciences Laboratory, Singapore*
[2] *Genome Institute of Singapore, Singapore*
[3] *Institute of Molecular and Cell Biology, Singapore*
[4] *Department of Biological Sciences, National University of Singapore, Singapore*
[5] *Computational Biology, Temasek Lifesciences Laboratory, Singapore*

**Correspondence to: Laszlo Orban, Reproductive Genomics Group, Temasek Lifesciences Laboratory, Singapore. E-mail: laszlo@tll.org.sg*

*Elia Stupka, Computational Biology Group, Temasek Lifesciences Laboratory, Singapore. E-mail: elia@tll.org.sg.*

*# These two authors contributed equally to the results published in the manuscript..*

Received: 4 May 2004
Revised: 25 June 2004
Accepted: 28 June 2004

### Abstract

**Studies on the zebrafish model have contributed to our understanding of several important developmental processes, especially those that can be easily studied in the embryo. However, our knowledge on late events such as gonad differentiation in the zebrafish is still limited. Here we provide an analysis on the gene sets expressed in the adult zebrafish testis and ovary in an attempt to identify genes with potential role in (zebra)fish gonad development and function. We produced 10 533 expressed sequence tags (ESTs) from zebrafish testis or ovary and downloaded an additional 23 642 gonad-derived sequences from the zebrafish EST database. We clustered these sequences together with over 13 000 kidney-derived zebrafish ESTs to study partial transcriptomes for these three organs. We searched for genes with gonad-specific expression by screening macroarrays containing at least 2600 unique cDNA inserts with testis-, ovary- and kidney-derived cDNA probes. Clones hybridizing to only one of the two gonad probes were selected, and subsequently screened with computational tools to identify 72 genes with potentially testis-specific and 97 genes with potentially ovary-specific expression, respectively. PCR-amplification confirmed gonad-specificity for 21 of the 45 clones tested (all without known function). Our study, which involves over 47 000 EST sequences and specialized cDNA arrays, is the first analysis of adult organ transcriptomes of zebrafish at such a scale. The study of genes expressed in adult zebrafish testis and ovary will provide useful information on regulation of gene expression in teleost gonads and might also contribute to our understanding of the development and differentiation of reproductive organs in vertebrates. Copyright © 2004 John Wiley & Sons, Ltd.**

**Keywords:** gonad; reproduction; ***Danio rerio***; fish; teleost; EST; cDNA; macroarray

**Supplementary material for this article can be found at http://www.interscience.wiley.com/jpages/1531-6912/suppmat**

### Introduction

During the past 30 years zebrafish (*Danio rerio*) has become one of the major vertebrate models for molecular genetics and developmental biology. The start of the Zebrafish Genome Project at the Sanger Center finally catapulted the species onto the platform of vertebrate genomics. The tool-set of zebrafish genomics — which includes an integrated genetic map based on four meiotic

Copyright © 2004 John Wiley & Sons, Ltd.

panels (e.g. Knapik *et al.*, 1998; Woods *et al.*, 2000) and two radiation hybrid panels (Geisler *et al.*, 1999; Hukriede *et al.*, 2001) among other tools — has been complemented with a genome assembly (**www.ensembl.org/Danio_rerio**), easing the task of those trying to decipher gene functions in zebrafish.

The analysis of expressed zebrafish sequences is still in the expansion phase. At the time of our 'data freeze' (at the end of January 2003, when the data were compared with those in GenBank), the number of zebrafish EST sequences in the dbEST database has exceeded 300 000 and several cDNA/oligonucleotide arrays (e.g. Clark *et al.*, 2001; Ton *et al.*, 2002) have become available during the past couple of years. On the other hand, a limited amount of data is available at present on the tissue-, organ- or developmental stage-specific transcriptomes of zebrafish. According to our knowledge, there are only three published reports on organ-specific EST data sets from zebrafish in the peer-reviewed literature: from embryonic heart (5102 ESTs; Ton *et al.*, 2000), from embryonic inner ear (18 000 ESTs; Coimbra *et al.*, 2002), and from adult gonads (1025 ESTs; Zeng and Gong, 2002).

Our knowledge about the genetic regulation of zebrafish reproduction is scarce. Sex chromosomes could not be identified in the zebrafish karyotypes (Pijnacker and Ferwerda, 1995; Sola and Gornung, 2001) and the molecular regulation of gonad differentiation is far from being understood. On the basis of a handful of studies the process seems to be complex, involving intense rearrangement from an ovary-like organ into the testis in males (Maack and Segner, 2003; Takahashi, 1977; Uchida *et al.*, 2002). Our primary interest is to understand the genetic regulation of the gonad differentiation process in zebrafish by using the tools of functional genomics.

Here we report on the analysis of adult zebrafish gonad transcriptomes and their comparison to that of the kidney, by computational and experimental tools in lieu of identifying genes potentially useful for the analysis of (zebra)fish gonad development and differentiation (see Figure 1 for the flowchart

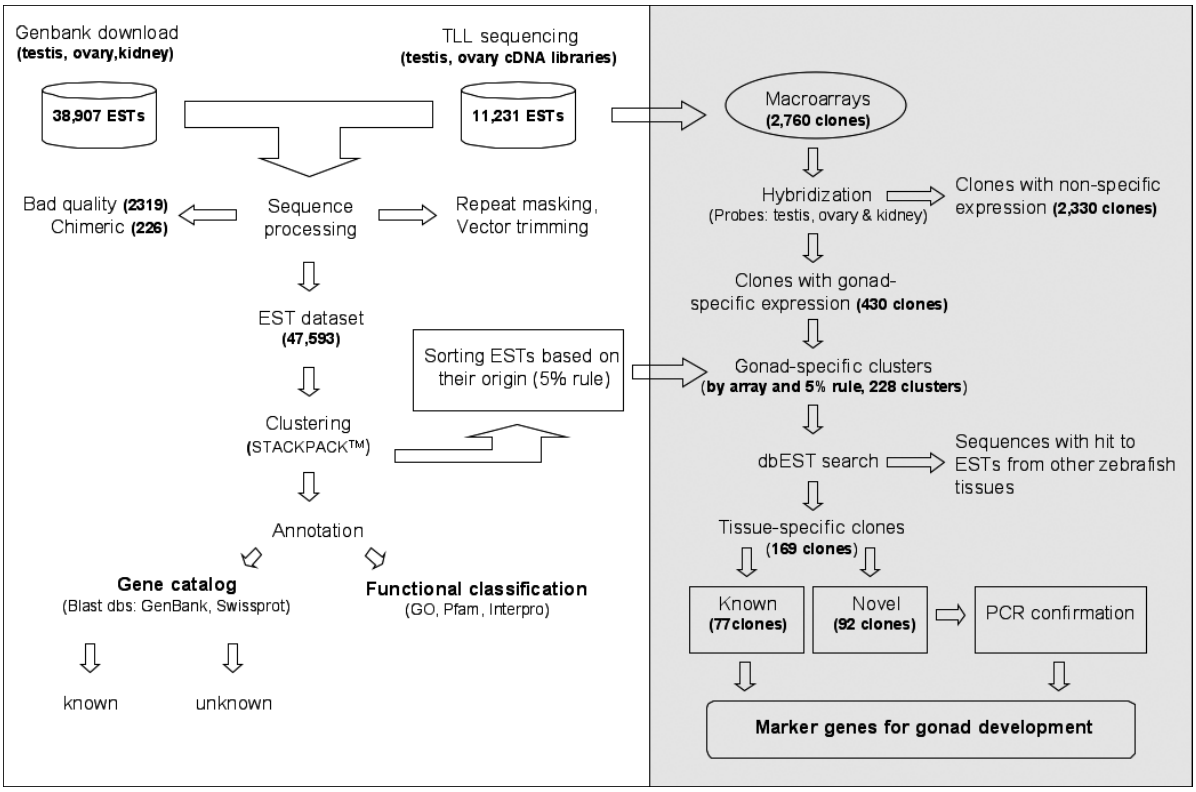

**Figure 1.** A flow chart depicting the order and connection of the experimental and computational procedures used of experimental and computational procedures used throughout the study).

### Materials and methods

### Fish stocks and sample collection

Zebrafish individuals from the AB strain and from a local strain, called Toh, were kept at our fish facility at ambient temperature and light cycle (12/12 h) in AHAB (Aquatic Habitats) recirculation systems. Sexually mature individuals of at least 3 months of age were anaesthetized in 0.04% 3-aminobenzoic acid ethyl-ester methanesulphonate (Sigma). The gonad (with the gonadal duct) and kidney were collected and transferred into ice-cold Trizol reagent (Gibco-BRL) and stored at −80 °C separately. For the generation of testis and kidney libraries, samples were pooled from 40–60 individuals, whereas ovary samples were combined from two to four individuals. Probes for the macroarray hybridization were generated from RNA isolated from testis and ovary collected from six different individuals, respectively, whereas the two kidney probes were pooled from two groups of six individuals containing both sexes.

As all of the gonad samples were isolated from fully mature individuals; they were expected to represent all germ cell types (i.e. from oogonia to fully mature oocyte in the ovary and from spermatogonia to spermatozoa in the testis) in addition to the somatic cells representative of the gonad type.

### RNA isolation and cDNA synthesis

Total RNA was isolated from the dissected tissues by Trizol (Gibco BRL) reagent according to the manufacturer's recommendation. Samples were treated with DNase (10 U in 100 µl volume; Roche) for 30 min at 37 °C, and the RNA was recovered by isopropyl-alcohol precipitation. The poly(A)$^+$ RNA fraction was isolated using oligo-dT cellulose chromatography (Stratagene). The quantity and integrity of the RNA was assessed by spectrophotometry and agarose gel electrophoresis, respectively. cDNA was synthesized from total RNA of Toh strain using SMART PCR cDNA synthesis kit (Clontech) according to the manufacturer's protocols.

### Construction of subtracted cDNA libraries

Three sets of subtractive hybridizations were performed: adult ovary (driver) from testis (tester), adult liver (d) from testis (t), and adult testis (d) from ovary (t). A PCR-Select™ cDNA subtraction kit (Clontech) was used to enrich for tissue-specific fragments from the SMART cDNA template (from Toh strain), according to the recommendations of the manufacturer. The selectively amplified cDNA fragments (in average 400–800 bp in length) from testis and ovary were ligated into pT-Advantage (Clontech) or to pGEM-T-Easy (Promega) cloning vector in order to generate subtracted libraries.

### Construction of full-length and normalized cDNA libraries

Full-length cDNA was synthesized from adult ovary and testis poly(A)$^+$ RNA (AB strain), respectively, with ZAP-cDNA Synthesis Kit (Stratagene), according to the manufacturer's protocols. Size-fractionated cDNAs (flanked by an *Eco*RI site at the 5′ end and an *Xho*I site at 3′ ends) were directionally cloned into Uni-ZAP XR vector and packaged using Gigapack Gold packaging extracts (Stratagene). The primary packaging mix was titrated and amplified to establish stable library stocks.

Phagemid particles were excised from the Uni-ZAP vector using ExAssist helper phage and SOLR strain according to the protocols (Stratagene). Excised pBluescript phagemids were used to infect *Escherichia coli* XL1-Blue cells and selected by ampicillin resistance and blue-white colour. White clones with cDNA inserts were randomly picked and grown overnight in LB-ampicillin culture.

Normalization of adult testis cDNA library was done by using a reassociation kinetics-based approach (Bonaldo *et al.*, 1996) with some modifications. Purified covalently closed single-stranded library DNA was produced *in vitro* by using GENETRAPPER cDNA Positive Selection System (GibcoBRL) according to the manufacturer's instructions. The resulting single-stranded circular DNA was purified from the remaining double-stranded plasmid by hydroxyapatite (HAP) chromatography (Bonaldo *et al.*, 1996).

PCR amplification of cDNA inserts was performed using the Expand High Fidelity PCR

Copyright © 2004 John Wiley & Sons, Ltd.

System (Roche) according to the manufacturer's instructions; 1 µl (2.5–5.0 ng) DNA template (a mixture of double-stranded plasmids from a cDNA library) was mixed with 2 µl dNTP stock (200 µM), 5 µl 20 µM solution of KS primer (5′-TCGAGGTC-GACGGTATC-3′), and 5 µl 20 µM SK primer (5′-CGCTCTAGAACTAGTGGATC-3′), 10 µl 10× Expand High Fidelity buffer, 0.75 µl Expand High Fidelity enzyme mix (2.6 units) and 76.25 µl water. The PCR mix was then overlaid with 50 µl mineral oil and subjected to the following amplification cycle conditions in a PTC 100 Thermocycler (MJ Research): 7 min while ramping up from room temperature to 94 °C; 20 cycles of 1 min at 94 °C, 2 min at 55 °C, and 3 min at 72 °C; and finally 7 min at 72 °C. PCR-amplified fragments were isolated by GFX columns (Amersham-Pharmacia) and dissolved in 5 µl TE. Then 3 µl (ca. 500 ng) PCR product was mixed with 7 µl (50 ng) library DNA (single-stranded circles prepared *in vitro* as described above), 15 µl deionized formamide, 1 µl (10 µg) 5′ blocking oligo (5′-GAGCTCCACCGCGGTGGCGGCCGC-TCTAGAACTAGTGGATCCCCCGGGCTGCA GGTTAACGGCACGAGG-3′) and 1 µl (10 µg) 3′ blocking oligo (5′-GTAATACGACTCACTATA-GGGCGAATTGGGTACCGGGCCCCCCCTC-GA-3′). This mixture was heated at 80 °C for 3 min under 10 µl mineral oil. Then 3 µl 10× buffer A (1.2 M NaCl, 0.1 M Tris pH 8.0 and 50 mM EDTA) were added, the hybridization was performed at 30 °C for 24 h. The remaining single-stranded circles were purified by HAP chromatography as described above, converted to double-strands using Sequenase version 2.0 (USB), and transformed into XL10-Gold competent cell (Stratagene).

### Generation of ORESTES libraries

ORESTES libraries were generated according to Neto *et al.* (1997, 2000). mRNA isolated from adult zebrafish testis was reverse-transcribed using oligonucleotide primers designed for amplified fragment length polymorphism (AFLP) analysis (Vos *et al.*, 1995) or for amplification of specific genes from *Arabidopsis*, yeast or rice. The amplification mastermix contained 5 µl 10× PCR buffer (Clontech) with 15 mM $MgCl_2$, 2 µl dNTP stock (10 mM each), 2 µl primer used for reverse transcription (10 µM), 1 U Advantage cDNA polymerase (Clontech), and 1 µl first strand cDNA. PCR conditions were: an initial cycle of 5 min at 94 °C, 2 min at 37 °C, 2 min at 72 °C followed by 35 cycles of 45 s at 94 °C, 1 min at 45 °C, and 1.5 min at 72 °C. The amplified product (10 µl) was checked on 2% agarose gel. PCR products with a single, predominant band reflecting the amplification of a highly abundant transcript were not processed further. The remaining amplification products with a smear or multiple bands (>500 bp) were then cloned into pGEM-T vector (Promega) and transformed into XL-10 Gold competent cell.

### Amplification and partial sequencing of cDNA inserts

White colonies were randomly picked and grown overnight in deep (2 ml) 96-well plates (Axygen) containing 1 ml LB-ampicillin medium; 100 µl O/N culture was mixed with an equal volume of glycerol and stored at −80 °C in 96-well tissue-culture plates. O/N culture (1 µl) was used directly for colony PCR reactions (25 µl final volume) with M13 forward (−20) and M13 reverse primers. PCR product (5 µl) was used for alkaline phosphatase and exonuclease I treatment: 0.2 µl 10× SAP buffer (USB) and 2 µl enzyme mix [containing 0.25 U shrimp alkaline phosphatase (USB) and 0.1 U exonuclease I (both from USB) were added] and 0.2 µl 10× SAP buffer were added and the samples were incubated at 37 °C for 30 min in order to eliminate PCR primers. The reaction mixture was then incubated at 80 °C for 15 min to inactivate the enzymes and then diluted with distilled water to 20 µl; 3 µl were used for cycle sequencing using BigDye Terminator v3.0 kit (Applied Biosystems) and M13 reverse primer. (In the directionally cloned full-length libraries, 5′ ends were sequenced. The orientation of the rest of the clones — subtracted and ORESTES libraries — was random.) The conditions for cycle sequencing were as follows: 50 °C for 1 min and 94 °C for 5 min, followed by 30 cycles of amplification (94 °C for 30 s, 50 °C for 15 s and 60 °C for 4 min) with 1 °C/sec ramping. Reaction products were precipitated by ethanol, dissolved in 20 µl distilled water and separated on an ABI 3700 capillary electrophoresis machine (Applied Biosystems).

### Sequence analysis and EST clustering

Sequences generated in our lab were cleaned from vector arms and adapters by using the Sequencher

Copyright © 2004 John Wiley & Sons, Ltd. 

4.05 software (Gene Codes Corp.) in manual mode. Zebrafish ESTs derived from adult testis, ovary and kidney cDNA libraries were downloaded from the dbEST division of GenBank (dataset from 11 September 2002) using the batch Entrez retrieval system (for details on the origin of clones, see Table A1 in the Supplementary Material). The public ESTs were combined with gonad ESTs generated in our laboratory (the GenBank IDs are in the following range: CO349711–CO360-835) and the whole dataset was subjected to a thorough cleaning procedure, consisting of trimming of vector arms, masking with RepeatMasker (**http://www.repeatmasker.org**) and removal of short (<100 bp) as well as low-quality (>3% N) sequences.

EST clustering and assembly was carried out using the STACKPACK™ clustering tool (Christoffels *et al.*, 2001; Miller *et al.*, 1999) on a HP-Compaq Alpha ES40 architecture. The d2-clustering step was executed with a word size of 6, a window size and minimum sequence size of 100 bases and a similarity threshold of 96%.

The combined gonad and kidney datasets were first clustered separately to check for presence of chimeras. The 18 biggest clusters were screened for chimeric sequences by searching with the consensus sequences in GenBank using BLAST (Altschul *et al.*, 1990, 1997) in two repeated steps and removing those for which the two ends clearly matched two different genes. The 20 biggest clusters of the kidney set were treated the same way. Altogether, 226 suspected chimeric ESTs were identified and removed (see Table A2 in the Supplementary Material for the list of GenBank-derived clones suspected to be chimeric).

The resulting final dataset was re-clustered as described above and used to construct a partial transcriptional profile for the testis, ovary and kidney of adult zebrafish. The proportion of ORF-containing sequences was determined by ESTScan (Iseli *et al.*, 1999) in both the clusters and singletons, respectively. Using the predicted ORFs, BLAST searches were carried out to identify putative homologues in Swissprot (**ftp.expasy.org**), TrEMBL (Boeckmann *et al.*, 2003) and NCBI's non-redundant protein database (**http://www.ncbi.nlm.nih.gov/BLAST/blast_databases.shtml**). For functional analysis the translated sequences obtained from ESTScan were annotated for protein domains and functional sites by matching them against the PFAM, PROSITE and PRINTS databases (Attwood *et al.*, 2003; Bateman *et al.*, 2002; Sigrist *et al.*, 2002). The annotated domains were assigned to Gene Ontology (GO) molecular function categories using mappings provided by the GO Consortium (Ashburner *et al.*, 2000).

For the phylogenetic analysis of ZP genes, the sequences were first aligned using CLUSTALW (Thompson *et al.*, 1994) and the trees constructed using the neighbour-joining method, using maximum likelihood distances (PHYLIP package; **http://evolution.genetics.washington.edu/phylip.html**). Bootstrapping was done using 1000 pseudosamples of the dataset.

### Generation and use of 'Gonad UniClone' macroarrays

In order to reduce redundancy, we re-arranged our clone set. Clones representing 1419 clusters and 1342 singletons have been selected from full-length or normalized libraries ('Gonad UniClone' set). Thirty 96-well plates were filled with colony PCR-amplified inserts from the selected clones.

Two macroarrays were produced by replicating this 'Gonad UniClone' set onto Hybond-N (Amersham-Pharmacia) nylon membranes in $4 \times 4$ arrangements, using a Biomek 2000 Workstation (Beckman). Each membrane contained empty vectors, and clones with viral and plant-derived inserts (negative controls) as well as cDNA fragments from 12 zebrafish housekeeping genes (positive controls). In the upper right corner (A1) of each and all the four corners of the first, fourth, thirteenth, and sixteenth plates the PCR product was replaced with 5 pg/µl DIG-labelled control DNA (Roche) to help orientation on the arrays after detection. DNA was then denatured (10 min, 1.5 M NaCl, 0.5 M NaOH), renatured (10 min, 1.5 M NaCl, 0.5 M Tris, pH 8.0) and linked to the membrane using UV light (120 mJ on both sides).

Testis-, ovary- and kidney-derived cDNA probes (see section on 'Fish stocks and sample collection' for detailed origin of probes) were generated by replacing half of the dNTP in the amplification step of the SMART cDNA synthesis with DIG-labelling dNTP mix (Roche). Excess of DIG-labelled dUTP was removed using GFX columns (Amersham-Pharmacia). Air-dried 'Gonad UniClone' macroarrays were pre-hybridized in 5 ml EasyHyb solution (Roche) at 50 °C for 2–3 h in a SI 20H

Copyright © 2004 John Wiley & Sons, Ltd. 

hybridization oven (Stuart Scientific). The solution was replaced with 5 ml fresh EasyHyb solution containing 0.3–0.6 μl probe (depending on the relative strength of the probe determined in a titration experiment) and hybridization was performed at the conditions listed above. Washing of membranes was conducted at 68 °C, 2 × 5 min with 40 ml buffer #1 (2× SSC, 0.1% SDS) and twice with 40 ml buffer #5 (0.05× SSC, 0.1% SDS) for 15 min each. Non-isotopic detection of the hybridized probe was conducted according to the Roche manual. Chemiluminescent signal (from dephosphorylated CPD-Star substrate) was recorded on BioMax ML film (Kodak) by taking multiple exposures for every membrane.

The best images were captured with FluorS-Multiimager (BioRad) and signal/background intensities were quantified using ImaGene 4.0 (BioDiscovery) software. Data was further processed in Microsoft Excel. After defining relative signal intensity for each spot on the separate membranes (signal median minus local background median), values were normalized across membranes based on the values measured from housekeeping genes. (Mean signal intensity for the 12 × 3 positive spots have been defined for each membrane, and also across membranes. Each intensity value from a given membrane was then multiplied by the quotient of the membrane average and the mean of the membrane averages.) A gene was considered to be expressed in a given tissue if the median of normalized values from six independent hybridizations exceeded the mean value of the negative controls plus twice their standard deviation (threshold = mean + 2 × SD of the negative controls). The expression of a clone was labelled as potentially tissue-specific when: (a) the median value for a clone in one tissue was higher than threshold, but from the other two tissues fell below that; and (b) there was a significant difference between the mean of the values from the given and the two other tissues (assessed by Student's $t$-test).

### Confirming the specificity of expression by PCR

In order to validate the tissue-specific genes obtained from the combinatorial 'wet-and-dry' approach, 45 such clones were selected and their expression pattern in adult zebrafish tissues were analysed. 'Smart cDNA' samples (Clontech) were generated from adult zebrafish testis, ovary, kidney and rest of body. The expression pattern of the selected genes was re-tested by PCR-amplification using specific primers designed (Primer Select, DNAStar) to their sequences and using the 'Smart cDNAs' as templates. The reaction mixtures contained the following in 12.5 μl total volume: 1.25 μl 10× reaction buffer, 50 μM dNTP mix, 5 pmol forward and reverse primers (see Table A6 in the Supplementary Material for full list of primers used), 10–90 ng template and 0.25 U Advantage cDNA polymerase (Clontech). As a positive control, 1 μl PCR-amplified insert from the appropriate cDNA clone was used for every primer pair. The thermal cycle profile consisted of an initial denaturation at 95 °C for 1 min, followed by 26 cycles of 94 °C for 10 s, annealing for 15 s and 68 °C for 1 min, and a final extension step of 68 °C for 3 min. 6 μl PCR product was separated on 2% agarose gel.

### Results

### Libraries and sequencing

We have generated four different kinds of cDNA libraries from adult zebrafish gonads for the isolation of testis- or ovary-derived clones: three subtracted libraries, two non-normalized and two normalized full-length libraries as well as 27 ORF expressed sequence tag (ORESTES) mini-libraries (see Table A1 in the Supplementary Material for complete list of sources used). Over 14 000 clones were picked randomly, their insert was amplified by colony PCR and end-sequenced from one direction (5′ or random, depending on the library of origin). The resulting sequences were trimmed, masked and cleaned by removing low-quality/short reads as well as repeat sequences, resulting in 7674 testis-derived and 2859 ovary-derived EST sequences.

We have also downloaded from the dbEST database nearly 10 000 testis-derived, over 15 000 ovary-derived and over 14 000 kidney-derived zebrafish EST sequences (Table A1 in the Supplementary Material). (Kidney was chosen as a somatic comparison, since clone sets in GenBank for all other major organs of adult zebrafish either contained a limited number of clones or originated from mixed sources.) Following the removal of 226 suspected chimeric EST sequences (see Table A2 in the Supplementary Material for suspected chimeric ESTs among public sequences), we

Copyright © 2004 John Wiley & Sons, Ltd. 

merged the public ESTs with the testis and ovary clone sets derived from our libraries to form a combined dataset with a total of 47 593 ESTs. This final dataset contained 16 479 testis-derived ESTs, 17 696 from the ovary and 13 418 from the kidney (Table 1).

### EST clustering

Gene indices — built by grouping together ESTs derived from the same transcript — provided us a picture on the unique and common transcripts expressed in the three organs. After masking the low complexity regions and repetitive elements in the sequences, we clustered the dataset. The consensus sequences for each of the clusters were then classified according to the tissue origin of their component ESTs. To account for the possibility of low-level contamination from other organs during the isolation process, clusters with at least 95% ESTs originating from a single organ were still considered as putative organ-specific ('5% rule').

A total of 402 clusters (incorporating 7569 ESTs) contained sequences from all three organs, 2132 clusters (14 879 ESTs) from two organs, whereas the remaining 3673 clusters (14 419 ESTs) from only one (Figure 2). The proportion of potentially organ-specific ESTs was similar for the three organs tested: testis, 19.4% of all ESTs (5038 unique sequences); ovary, 17.6% (3939); and kidney, 15.8% (5422).

All 'non-GenBank' singletons derived from our study and those clusters without a single GenBank-derived EST were BLAST-searched against the proteins and ESTs present in GenBank at the time of the 'data-freeze' (January 2003). Of the 2845 singletons that were of acceptable quality, 1068 had no hits, whereas 125 of the 477 clusters did not find a similar sequence in GenBank. Therefore, we have added novel sequence information on 1193 zebrafish transcripts to the public database.

**Table 1.** Generation of the final EST dataset from adult zebrafish testis, ovary and kidney

| | Testis | Ovary | Kidney | Total |
|---|---|---|---|---|
| Sequences | 17 726 | 18 382 | 14 034 | 50 142 |
| Removed* | 1204 | 528 | 591 | 2 323 |
| Chimeric | 43 | 158 | 25 | 226 |
| Final EST set | 16 479 | 17 696 | 13 418 | 47 593 |

* Low quality sequences, repeat sequences or <100 bp following masking.

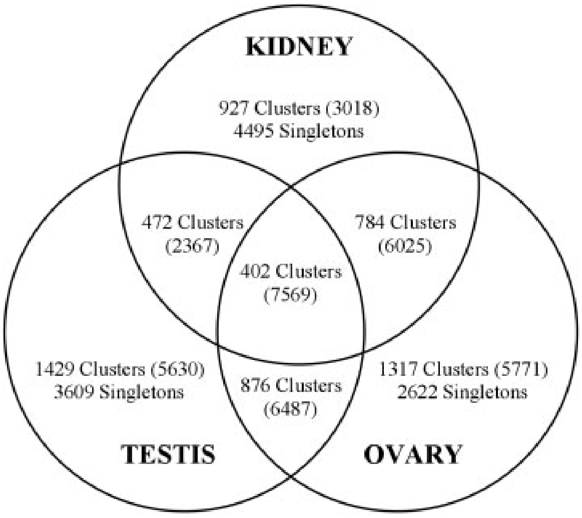

**Figure 2.** Distribution of clusters and singletons according to the origin of the EST sequences. Following the removal of suspected chimeric sequences, the resulting final EST dataset containing sequences both from GenBank and our laboratory was subjected to a thorough cleaning procedure. EST clustering and assembly was carried out using the STACKPACK™ clustering tool, producing a transcriptional profile for the testis, ovary and kidney of adult zebrafish. The clusters were classified according to the tissue origin of their component ESTs. To account for the possibility of low-level contamination from other organs during the isolation process, clusters with at least 95% ESTs originating from a single organ were still considered as putative organ-specific ('5% rule'). See Materials and methods for additional details

### Sequence analysis

Single pass ESTs are error-prone and often contain artifacts such as genomic DNA contamination (Hillier *et al.*, 1996). We used the combination of ESTScan (Iseli *et al.*, 1999) and BLAST (Altschul *et al.*, 1990, 1997) to evaluate the gene content of our sequences (see Materials and methods for details). About 62% of the total number of sequences — from all three organs combined — had a significant sequence similarity with a known gene or protein (with an e-value of less than $1e^{-5}$), and the vast majority of these contained a predicted ORF (Table 2).

Among the unknown genes (no significant sequence similarity to any of the databases) the percentage of ORF-containing sequences was higher

Copyright © 2004 John Wiley & Sons, Ltd. 

**Table 2.** Analysis of sequences (clusters and singletons) in the final dataset by BLAST and ESTScan

| | Testis | Ovary | Kidney |
|---|---|---|---|
| Total | 6788 | 6001 | 7080 |
| ORF-containing | (83.8%) | (85.8%) | (78.7%) |
| Known* | 4078 | 3991 | 4187 |
| ORF-containing | (98.1%) | (98.1%) | (97.1%) |
| Unknown** | 2710 | 2010 | 2893 |
| ORF-containing | (62.4%) | (61.4%) | (52.0%) |

* Known: singletons and clusters that show a significant BLAST match (at least an e-value of $1e^{-5}$) to Swissprot, TrEMBL, NCBI's non-redundant protein or NCBI's non-redundant nucleotide data set.
** Unknown: those without a significant BLAST match in the testis (62.4%) and ovary (61.4%) than in the kidney (52.0%) (Table 2).

### The most abundant ESTs from adult zebrafish testis, ovary and kidney

We analysed the distribution of contributing ESTs between testis, ovary and kidney in the 100 biggest clusters of the final dataset (Table A3 in the Supplementary Material). The majority of these comprised of ESTs from at least two organs and showed similarity to known genes, e.g. elongation factor $1\alpha$, $\beta$-actin or $\beta$-tubulin. In contrast, there were 24 clusters with ESTs derived from a single organ (5% rule applied). Five of these clusters contained testis-derived sequences, 18 constituted ovary-derived ones, whereas the remaining one contained exclusively kidney-derived ESTs (Table A3 in the Supplementary Material).

Twenty-two consensus sequences encoded for zebrafish orthologues of genes with functions related to reproduction in other organisms, e.g. prostaglandin E synthase (Jakobsson *et al.*, 1999), rhamnose-binding lectin (Tateno *et al.*, 1998) or zygote arrest 1 (Wu *et al.*, 2003). On the other hand, the list also included several genes (e.g. dihydropteridine reductase, septin, dim1p homologue and cystein proteinase) for which enhanced expression in the gonad has not been described previously.

### Functional classification of the clusters

The translated sequences obtained from ESTScan were annotated for protein domains and functional sites. Domains were then assigned to GO molecular function categories and the relative proportions of these categories were compared in the testis, ovary and kidney. The frequency of the 100 most frequent domains in the three organs, together with their GO categories, is shown in Table A4 in the Supplementary Material.

Surprisingly, the overall domain distribution was very similar for all three organs. Nearly half of the clusters fell into the category of unknown molecular functions, whereas the second and third most populous groups were 'binding activity' and 'enzyme activity' (Table 3; see Figure 3A for a typical result).

The categories of 'binding activity' and 'enzyme activity' were then analysed in further detail by assigning their genes into more specific subcategories for all three organs (Figure 3B). At this level more differences were found. Testis had fewer genes with 'transferase activity', than the other two organs. Ovary, on the other hand, had more genes with 'carbohydrate-binding activity', probably due to high-level expression of the rhamnose-binding lectins. Both gonads had many more genes with 'nucleic acid binding ability' and less with 'lyase ability' than the kidney.

**Table 3.** Comparison on the molecular functions of genes in testis-, ovary- and kidney-derived EST clusters

| Molecular function* | Testis (%) | Ovary (%) | Kidney (%) |
|---|---|---|---|
| Unknown | 48.59 | 44.26 | 48.77 |
| Binding activity | 18.51 | 20.12 | 18.11 |
| Enzyme activity | 14.35 | 15.95 | 15.16 |
| Transporter activity | 4.08 | 5.08 | 4.09 |
| Signal transduction activity | 3.85 | 4.14 | 4.40 |
| Transcription regulator activity | 2.99 | 3.53 | 3.10 |
| Structural molecule activity | 2.75 | 2.59 | 1.72 |
| Enzyme regulator activity | 1.39 | 1.50 | 1.22 |
| Motor activity | 0.84 | 0.20 | 0.33 |
| Chaperone activity | 0.80 | 0.62 | 0.56 |
| Translation regulator activity | 0.60 | 0.67 | 1.08 |
| Defence/immunity protein activity | 0.36 | 0.25 | 0.41 |
| Toxin activity | 0.35 | 0.54 | 0.27 |
| Apoptosis regulator activity | 0.26 | 0.42 | 0.38 |
| Cell adhesion molecular activity | 0.15 | 0.07 | 0.29 |
| Nutrient reservoir activity | 0.07 | 0.02 | 0.02 |
| Anticoagulant activity | 0.04 | 0.00 | 0.02 |
| Protein tagging activity | 0.04 | 0.02 | 0.03 |
| Antioxidant activity | 0.00 | 0.02 | 0.03 |
| Cytoskeletal regulator activity | 0.00 | 0.00 | 0.02 |

* Molecular functions were assigned to Gene Ontology molecular function categories using mappings provided by the GO Consortium (Ashburner *et al.*, 2000).

Copyright © 2004 John Wiley & Sons, Ltd. 

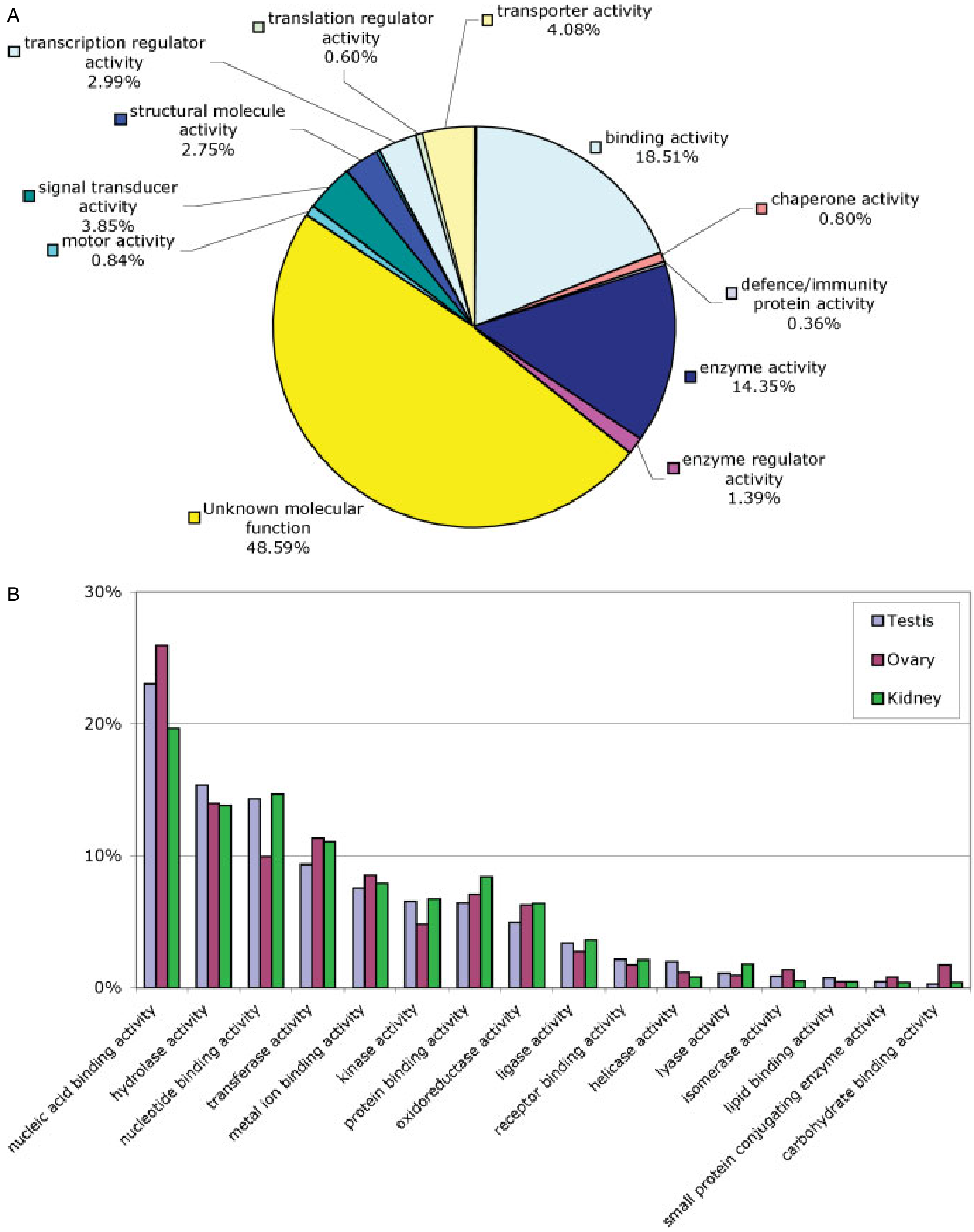

**Figure 3.** Domain distributions in the adult zebrafish testis, ovary and kidney. The translated sequences obtained from ESTScan were annotated for protein domains and functional sites. The annotated domains were assigned to GO molecular function categories, using mappings provided by the GO Consortium, and the relative proportion of the functional categories in the testis, ovary and kidney were compared. (A) GO molecular functions of clusters specific to testis (pie charts for ovary- and kidney-specific domains are very similar); (B) detailed classification of the categories of 'binding activity' and 'enzyme activity', comparing the relative proportions in the three organs

Copyright © 2004 John Wiley & Sons, Ltd. 

### Identification of novel genes with testis- and ovary-specific expression

Experimental and computational tools were applied in succession for the identification of genes with gonad-specific expression in adults (Figure 1). Two 'Gonad UniClone' macroarrays were produced, they contained adult testis- and ovary-derived full-length cDNA clones. The 2761 clones spotted onto the two membranes were selected from our cDNA collection on the basis of the *'in silico'* normalization results. Three different kinds of cDNA probes (from adult testis, adult ovary and adult kidney) were hybridized onto the membranes in six parallels for each organ. The resulting patterns were analysed and compared to each other (see Figure 4 for typical examples of hybridization patterns).

Clones showing significant signal with one of the gonad probes, but not with the other two, were considered as potentially testis- or ovary-specific, and matched to clusters to identify a unique clone set. Clusters with more than 5% ESTs from the other two organs were removed from the dataset. The consensus sequence of the remaining clusters was then used to search the dbEST database in GenBank to eliminate those clones, which show clear homology to ESTs derived from any other adult zebrafish organ, leaving 169 clones (Figure 1; Table 4). A total of 77 of these clones were with known functions and some (e.g. histone 2A, piwi and tektin 1) with gonad-specific or gonad-enhanced expression in other organisms (Table A5 in the Supplementary Material). The rest were novel genes: 53 with potentially testis-specific and 39 with potentially ovary-specific expression patterns in the adult zebrafish (Table 4).

The expression pattern of 45 novel clones (mostly those with potential orthologues with unknown function in other vertebrate classes) was re-tested by PCR-amplification analysis using specific PCR primers and templates from a cDNA

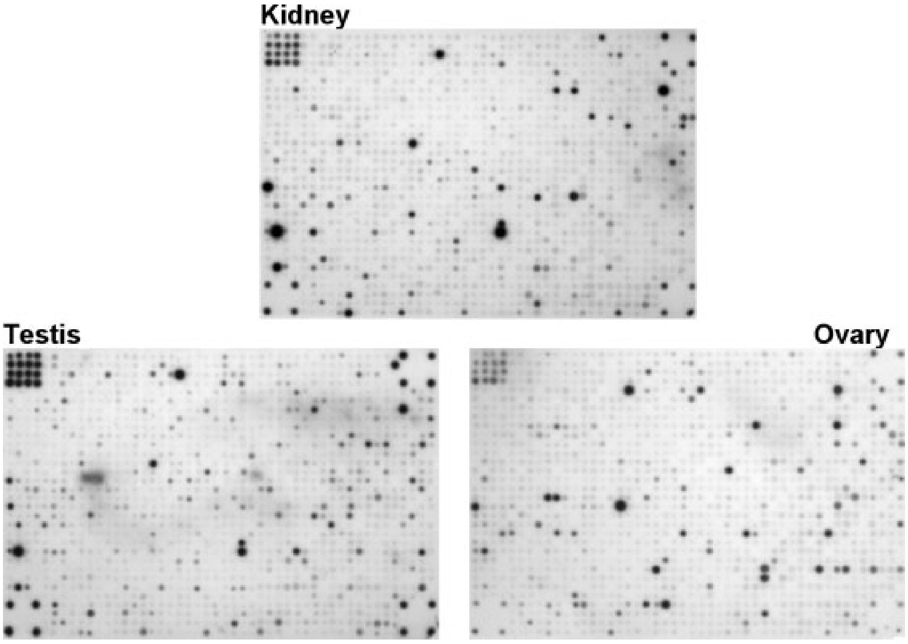

**Figure 4.** Typical results from 'Gonad UniClone' macroarrays hybridized with three different kinds of organ-derived, DIG-labelled probes. Two macroarrays were produced by replicating our 'Gonad UniClone' cDNA set containing adult zebrafish testis- or ovary-derived, PCR-amplified cDNA inserts from full-length cDNA library onto nylon membranes in a $4 \times 4$ arrangement with the appropriate controls. Following hybridization with digoxigenin-labelled organ-derived cDNA probes, washing and non-isotopic detection of the hybridized probe chemiluminescent signal was recorded on film. Relative signal intensity values were normalized across membranes based on the values measured from housekeeping genes. A gene was considered to be expressed in a given tissue if the median of normalized values from six independent hybridizations exceeded the mean value of the negative controls plus twice their standard deviation. The type of probe is indicated at one of the upper corners of each image

Copyright © 2004 John Wiley & Sons, Ltd. 

**Table 4.** Organ-specific clones identified by subsequent application of 'wet and dry' genomic tools (differential hybridization on cDNA array, *in silico* subtraction and BLAST analysis)

| | Testis | Ovary |
|---|---|---|
| Clones on array derived from the organ | 1748 | 1012 |
| Organ-specific hybridization | 118 | 312 |
| *In silico* subtraction* | 93 | 135 |
| No BLAST hit in other adult organ** | 72 | 97 |
| Novel | 53 | 39 |

* The sum of clusters and singletons specific to a given organ ('5% rule' applied).
** BLASTed against dbEST and removed those with matching sequence to an EST originating from any non-gonadal adult organ.

panel, containing samples isolated from adult zebrafish testis, ovary, kidney and rest-of-body (Figure 5). Primer pairs for seven clones amplified a product only from the positive control, but not from any of the organ-derived cDNAs, whereas eight were expressed in each sample tested. The remaining 30 reactions all showed gonad-enhanced or gonad-specific expression: 15 expression patterns were testis-specific, whereas six of them ovary-specific. The rest showed testis-enhanced (six clones) or ovary-enhanced (three clones) expression pattern, with strong product from one of the two gonads and weak one from at least one additional organ. Therefore, the results of the PCR assay confirmed organ-specificity for 21 of the 45 clones analysed.

### Discussion

The catalogue of genes expressed in a given organ, tissue or cell type at a particular developmental stage (the transcriptome) is important for molecular biologists for several reasons. It helps to identify the gene sets transcribed in the selected organ (tissue, cell), allowing for better understanding of molecular processes and their genetic regulation by using cDNA arrays. EST collections and clustered cDNA sets — especially those with full-length sequence — produced from these clones also have an important role in 'complementing and advancing identification of genes from annotated genome sequences' (Wakimoto, 2000). The progressing sequencing of the zebrafish genome at the Sanger Center has reached a 5.3× coverage and provided the researchers with the third assembly, which contains over 58 000 supercontigs, covering about 86% of the genome.

Our paper describes the analysis of partial transcriptomes of the adult zebrafish gonads by comparing clustered cDNA sets (based on an average of 15 000 ESTs/organ) from testis and ovary to

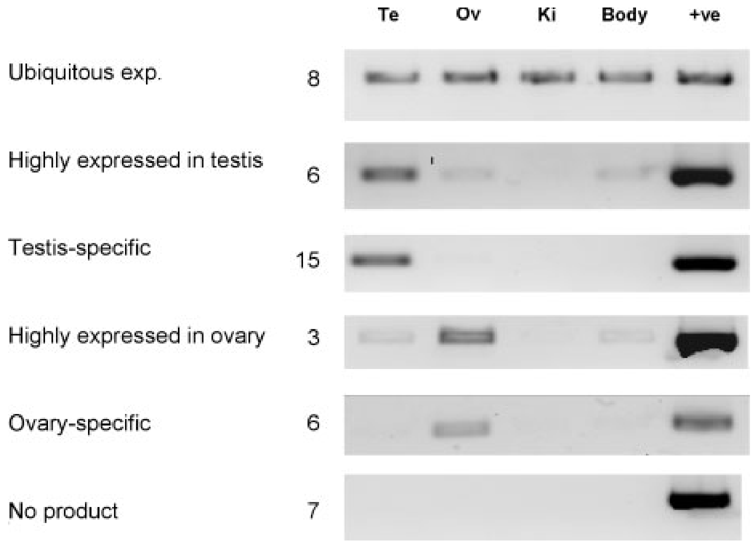

**Figure 5.** The expression pattern of a selected set of potentially organ-specific genes analysed by PCR amplification by using cDNA panel generated from total RNAs isolated from adult organs as template. Labels for templates: Te, testis; Ov, ovary; Ki, kidney; Body, rest of body (all organs, except gonad and kidney); +ve, PCR-amplified cDNA insert from the clone in question (used as positive control)

Copyright © 2004 John Wiley & Sons, Ltd. 

each other and to a somatic control (kidney). One-third of the gonad-derived ESTs used in the study were produced in our laboratory, the rest were obtained from GenBank. BLAST and ESTScan analysis of the clustered set of sequences from the three organs yielded very similar results, with the exception of the ratio of ORF-containing sequences among 'unknown genes', which was higher in the two gonads than in the kidney.

Our effort to identify genes with gonad-specific expression from 2760 cloned inserts spotted onto our 'Gonad UniClone' cDNA array was based on combining the power of experimental and computational genomic tools. Stepwise application of an experimental and three computational methods allowed us to select 72 clones with potentially testis-specific and 97 clones with potentially ovary-specific expression. Over 45% of these clones either show similarity to hypothetical genes from other vertebrates or are without a BLASTx hit in GenBank. A subset of 45 clones (all with unknown function) was analysed by PCR amplification from organ-specific cDNA templates. The results confirmed that the expression for nearly half of them is restricted either to the adult testis (15 clones) or the ovary (six clones). These genes will be useful as markers for the adult gonad in gene expression studies. Those with testis-specific expression in adults will be subjected to detailed analysis to select the ones with early expression in the differentiating testis. Currently the males can only be identified from dissected samples either by histology (Maack and Segner, 2003), or by the phenotype of the dissected gonad 5 weeks post-fertilization (wpf; R.B., unpublished data). Although stable transgenic zebrafish lines with the enhanced expression of EGFP-containing reporter constructs in the ovary have been reported (Hsiao and Tsai, 2003; Onichtchouk *et al.*, 2003), they can only be used to identify the males following 5–6 wpf. The reason for this is that most individuals seem to pass through an early phase, where their gonad would exhibit female-like expression pattern (Hsiao and Tsai, 2003; Takahashi, 1977). The availability of markers with an early testis-specific expression pattern would likely advance the study of the gonad differentiation process in zebrafish and possibly in related teleost species as well.

Assigning genes to functional categories by using the criteria provided by the GO Consortium helps with the understanding of their potential function, which in turn eases the task of explaining differences among the gene sets (co-)expressed in various organs. At the GO functional level, the domain distributions among the sequences derived from zebrafish testis, ovary or kidney are nearly identical and similar to GO pie charts produced from genes expressed specifically in the mouse testis (Bono *et al.*, 2003). However, they are different from that of mouse kidney (Bono *et al.*, 2003), as the latter contains substantially more 'enzymes' and 'transporters', than the mouse testis or any of the three zebrafish organs studied by us. The reason for this difference could be a relatively low number of clones (67) used for the generation of the mouse kidney GO pie chart. Differences among the three fish organs in the size of 'nucleic acid binding activity' group might point at increased level of transcription in the gonads, a fact well known for certain cell types in the testis (Kleene, 2001), but not for those of the ovary.

The analysis of the 100 biggest clusters present on the TOK (testis–ovary–kidney) EST set provided interesting data. The most abundantly expressed groups of sequences in our final dataset are those of the zona pellucida proteins (ZPs; Bleil and Wassarman, 1980b). These sulphated glycoproteins are the main constituents of the enveloping layer surrounding vertebrate eggs (Wassarman *et al.*, 1999), acting as primary and secondary sperm receptors in oocytes (Bleil and Wassarman, 1980a, 1983). According to a recently revised classification, there are four ZP subfamilies in vertebrates: ZPA, ZPX, ZPB and ZPC (Spargo and Hope, 2003). Fish genomes usually have a variable number of ZPB and ZPC genes (Del Giacco *et al.*, 2000; Kanamori *et al.*, 2003), and they are expected to contain at least one ZPX gene (Kanamori *et al.*, 2003; Spargo and Hope, 2003). We performed a phylogenetic analysis on the clusters that matched a ZP gene, along with a subset of the sequences listed by Spargo and Hope (2003; see Table A7 in the Supplementary Material for the list). The topology of the resulting consensus tree (Figure 6) is nearly identical to that described by Spargo and Hope (2003). It also shows that among the ZP-homologous clusters in our dataset, one of them, TOK888, lies within the ZPX subfamily. On the other hand, no ZPA gene has been identified here or from other fish species previously (Spargo and Hope, 2003).

Copyright © 2004 John Wiley & Sons, Ltd. 

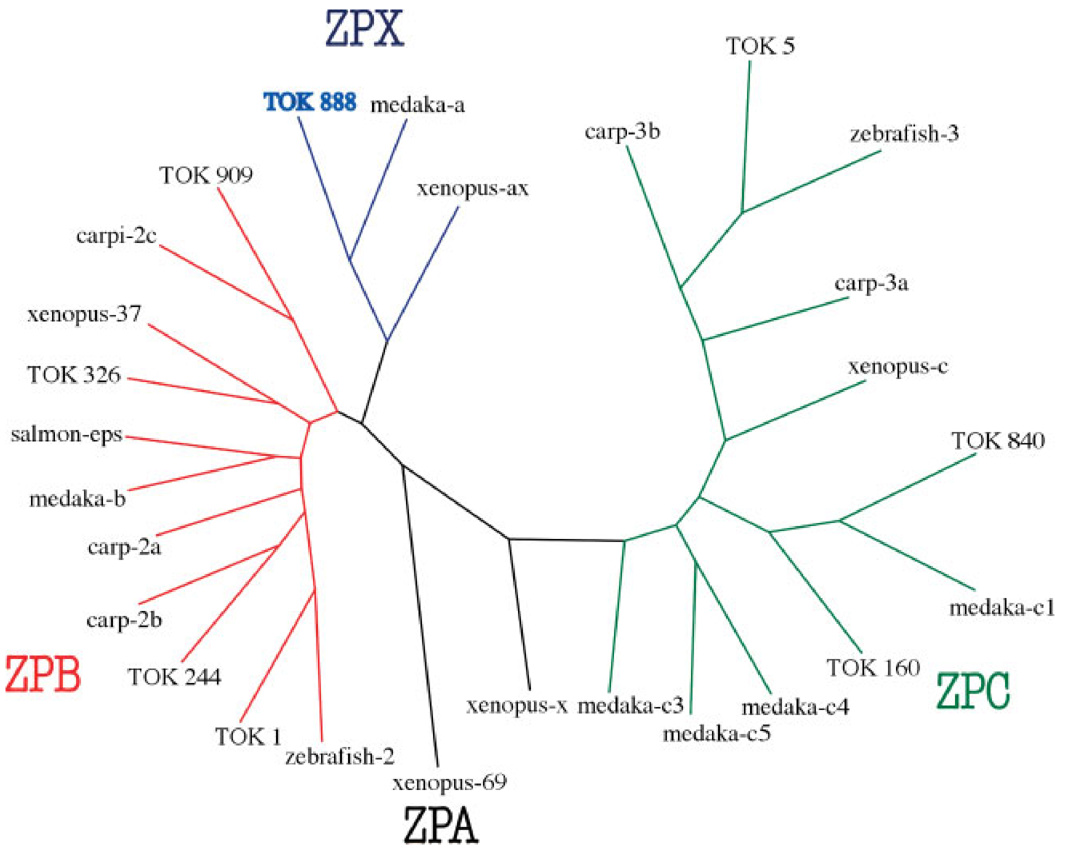

**Figure 6.** Phylogenetic tree of the zona pellucida (*zp*) gene family, updated by including several clusters from our dataset (labelled with TOK). All other sequences are from Spargo and Hope (2003)

Sequences coding for sugar-binding proteins, called lectins (for reviews, see Kilpatrick, 2002; Loris, 2002) are among the biggest clusters in our final dataset, showing ovary-specific expression. Several forms of rhamnose-binding lectins have been described from the eggs of steelhead trout (Tateno *et al*., 1998, 2001) and other fish species (e.g. Hosono *et al*., 1999; Tateno *et al*., 2002). Their main physiological role is thought to be protection of the embryos/larvae against pathogens (review: Ewart *et al*., 2001). C-type lectins and pentraxins are also present in large numbers among ovary-derived zebrafish ESTs, and they are implicated in defence mechanisms of vertebrates (Arason, 1996). Lectins are also expected to be involved in fertilization and embryonic developmental processes, as observed in sea urchin (Ozeki *et al*., 1995) and in intracellular transport within cells (Hauri *et al*., 2000, 2002). In fish they might also have a role in blocking polyspermy (Murata *et al*., 2000; Yasumasu *et al*., 2000).

Our 'Gonad UniClone' array is the second specialized zebrafish cDNA array — following that of Ton *et al*. (2002) — containing clones isolated from one organ type only, in our case the adult gonads. The use of such organ-derived arrays permits more efficient analysis of the target organ(s) due to higher coverage of their transcriptome than that offered by the general arrays (e.g. Clark *et al*., 2001; Lo *et al*., 2003). We used EST clustering to decrease the redundancy of our original dataset: in addition to the singletons, only a single clone from each cDNA cluster was spotted onto the 'Gonad UniClone' macroarray. In the absence of full-length cDNA sequences for most of our clones, we were unable to determine the exact redundancy of our spotted dataset. However, preliminary data from their 3′ ESTs (E. Low, personal communication) indicates redundancy value below

Copyright © 2004 John Wiley & Sons, Ltd. 

5%, which would in turn suggest the presence of over 2600 unique clones on the two membranes. The 'Gonad UniClone' cDNA set will be extended to contain approximately 7000–8000 full-length gonad-derived cDNA clones and converted into microarrays (in progress). We expect the 'Gonad UniClone' microarrays to be useful for the analysis of gene sets expressed in gonads, as demonstrated by others in *C. elegans* (Jiang *et al.*, 2001; Reinke *et al.*, 2000), *D. melanogaster* (Andrews *et al.*, 2000), mouse (Rockett *et al.*, 2001) and human (Schummer *et al.*, 1999).

Infertility is causing a problem for 10–15% of human couples (De Kretser and Baker, 1999; Maduro and Lamb, 2002) and genetic factors rank highly among the possible reasons (Lilford *et al.*, 1994). To date, knockout mouse has been used exclusively as a model system for studying mutations implicated in human reproductive disorders (Cooke and Saunders, 2002; Matzuk and Lamb, 2002). Among the testis- and ovary-specific zebrafish genes identified in our screen, over 30 have shown a high level of similarity to hypothetical genes, cDNAs or proteins described from the two sequenced mammalian genomes. The corresponding human and mouse orthologues of such genes — due to their conserved sequence and gonad-related function in vertebrates — might have a potential importance for the study of mammalian gonad physiology.

### Acknowledgements

The authors thank Professor Warren J. Ewens for advice on statistical analysis of the data; Balamurugan Kumarasamy, James Quek and Yina Cai for technical assistance; Elijah Low for preliminary information on unpublished data; and Drs Wei-Cai Yang, Zhong-Cao Yin, Naweed Naqvi, Jimmy Kwang and Mohan Balasubramanian for primers used for the amplification of ORESTES clones. The help of TLL Sequencing Facility is also acknowledged. This work was supported by research grants from the Temasek Lifesciences Laboratory and the Agency for Science, Technology and Research (A*STAR).

### References

Altschul SF, Gish W, Miller W, Myers EW, Lipman DJ. 1990. Basic local alignment search tool. *J Mol Biol* **215**: 403–410.

Altschul SF, Madden TL, Schaffer AA, *et al.* 1997. Gapped BLAST and PSI-BLAST: a new generation of protein database search programs. *Nucleic Acids Res* **25**: 3389–3402.

Andrews J, Bouffard GG, Cheadle C, *et al.* 2000. Gene discovery using computational and microarray analysis of transcription in the *Drosophila melanogaster* testis. *Genome Res* **10**: 2030–2043.

Arason GJ. 1996. Lectins as defence molecules in vertebrates and invertebrates. *Fish Shellfish Immunol* **6**: 277–289.

Ashburner M, Ball CA, Blake JA, *et al.* 2000. Gene ontology: tool for the unification of biology. *Nature Genet* **25**: 25–29.

Attwood TK, Bradley P, Flower DR, *et al.* 2003. PRINTS and its automatic supplement, prePRINTS. *Nucleic Acids Res* **31**: 400–402.

Bateman A, Birney E, Cerruti L, *et al.* 2002. The Pfam protein families database. *Nucleic Acids Res* **30**: 276–280.

Bleil JD, Wassarman PM. 1980a. Mammalian sperm-egg interaction: identification of a glycoprotein in mouse egg zonae pellucidae possessing receptor activity for sperm. *Cell* **20**: 873–882.

Bleil JD, Wassarman PM. 1980b. Structure and function of the zona pellucida: identification and characterization of the proteins of the mouse oocyte's zona pellucida. *Dev Biol* **76**: 185–202.

Bleil JD, Wassarman PM. 1983. Sperm-egg interactions in the mouse: sequence of events and induction of the acrosome reaction by a zona pellucida glycoprotein. *Dev Biol* **95**: 317–324.

Boeckmann B, Bairoch A, Apweiler R, *et al.* 2003. The Swiss-Prot protein knowledgebase and its supplement TrEMBL in 2003. *Nucleic Acids Res* **31**: 365–370.

Bonaldo MDF, Lennon G, Soares MB. 1996. Normalization and subtraction: two approaches to facilitate gene discovery. *Genome Res* **6**: 791–806.

Bono H, Yagi K, Kasukawa T, *et al.* 2003. Systematic expression profiling of the mouse transcriptome using RIKEN cDNA microarrays. *Genome Res* **13**: 1318–1323.

Christoffels A, van Gelder A, Greyling G, *et al.* 2001. STACK: Sequence tag alignment and consensus knowledgebase. *Nucleic Acids Res* **29**: 234–238.

Clark MD, Hennig S, Herwig R, *et al.* 2001. An oligonucleotide fingerprint normalized and expressed sequence tag characterized zebrafish cDNA library. *Genome Res* **11**: 1594–1602.

Coimbra RS, Weil D, Brottier P, *et al.* 2002. A subtracted cDNA library from the zebrafish (*Danio rerio*) embryonic inner ear. *Genome Res* **12**: 1007–1011.

Cooke HJ, Saunders PT. 2002. Mouse models of male infertility. *Nature Rev Genet* **3**: 790–801.

De Kretser DM, Baker HW. 1999. Infertility in men: recent advances and continuing controversies. *J Clin Endocrinol Metab* **84**: 3443–3450.

Del Giacco L, Diani S, Cotelli F. 2000. Identification and spatial distribution of the mRNA encoding an egg envelope component of the Cyprinid zebrafish, *Danio rerio*, homologous to the mammalian ZP3(ZPC). *Dev Genes Evol* **210**: 41–46.

Ewart KV, Johnson SC, Ross NW. 2001. Lectins of the innate immune system and their relevance to fish health. *Ices J Marine Sci* **58**: 380–385.

Geisler R, Rauch GJ, Baier H, *et al.* 1999. A radiation hybrid map of the zebrafish genome. *Nature Genet* **23**: 86–89.

Hauri HP, Appenzeller C, Kuhn F, Nufer O. 2000. Lectins and traffic in the secretory pathway. *FEBS Lett* **476**: 32–37.

Hauri HP, Nufer O, Breuza L, Ben Tekaya H, Liang L. 2002. Lectins and protein traffic early in the secretory pathway. In *Glycogenomics: The Impact of Genomics and Informatics*

Copyright © 2004 John Wiley & Sons, Ltd. 

*on Glycobiology*, Drickamer K, Dell A (eds), Portland Press: London; 73–82.

Hillier L, Lennon G, Becker M, *et al*. 1996. Generation and analysis of 280 000 human expressed sequence tags. *Genome Res* **6**: 807–828.

Hosono M, Ishikawa K, Mineki R, *et al*. 1999. Tandem repeat structure of rhamnose-binding lectin from catfish *(Silurus asotus)* eggs. *Biochim Biophys Acta Gen Sub* **1472**: 668–675.

Hsiao CD, Tsai HJ. 2003. Transgenic zebrafish with fluorescent germ cell: a useful tool to visualize germ cell proliferation and juvenile hermaphroditism *in vivo*. *Dev Biol* **262**: 313–323.

Hukriede N, Fisher D, Epstein J, *et al*. 2001. The LN54 radiation hybrid map of zebrafish expressed sequences. *Genome Res* **11**: 2127–2132.

Iseli C, Jongeneel CV, Bucher P. 1999. ESTScan: a program for detecting, evaluating, and reconstructing potential coding regions in EST sequences. In *Proceedings of the Seventh International Conference on Intelligent Systems for Molecular Biology*. Lengauer T, Schneider R, Bork P *et al.* (eds). Heidelberg, Germany, pp. 138–148.

Jakobsson PJ, Thoren S, Morgenstern R, Samuelsson B. 1999. Identification of human prostaglandin E synthase: a microsomal, glutathione-dependent, inducible enzyme, constituting a potential novel drug target. *Proc Natl Acad Sci USA* **96**: 7220–7225.

Jiang M, Ryu J, Kiraly M, *et al*. 2001. Genome-wide analysis of developmental and sex-regulated gene expression profiles in *Caenorhabditis elegans*. *Proc Natl Acad Sci USA* **98**: 218–223.

Kanamori A, Naruse K, Mitani H, Shima A, Hori H. 2003. Genomic organization of ZP domain containing egg envelope genes in medaka (*Oryzias latipes*). *Gene* **305**: 35–45.

Kilpatrick DC. 2002. Animal lectins: a historical introduction and overview. *Biochim Biophys Acta Gen Sub* **1572**: 187–197.

Kleene KC. 2001. A possible meiotic function of the peculiar patterns of gene expression in mammalian spermatogenic cells. *Mech Dev* **106**: 3–23.

Knapik EW, Goodman A, Ekker M, *et al*. 1998. A microsatellite genetic linkage map for zebrafish (*Danio rerio*). *Nature Genet* **18**: 338–343.

Lilford R, Jones AM, Bishop DT, Thornton J, Mueller R. 1994. Case-control study of whether subfertility in men is familial. *Br Med J* **309**: 570–573.

Lo J, Lee SC, Xu M, *et al*. 2003. 15000 unique zebrafish EST clusters and their future use in microarray for profiling gene expression patterns during embryogenesis. *Genome Res* **13**: 455–466.

Loris R. 2002. Principles of structures of animal and plant lectins. *Biochim Biophys Acta Gen Sub* **1572**: 198–208.

Maack G, Segner H. 2003. Morphological development of the gonads in zebrafish. *J Fish Biol* **62**: 895–906.

Maduro MR, Lamb DJ. 2002. Understanding new genetics of male infertility. *J Urol* **168**: 2197–2205.

Matzuk MM, Lamb DJ. 2002. Genetic dissection of mammalian fertility pathways. *Nature Cell Biol* **4**(suppl): s41–49.

Miller RT, Christoffels AG, Gopalakrishnan C, *et al*. 1999. A comprehensive approach to clustering of expressed human gene sequence: the sequence tag alignment and consensus knowledge base. *Genome Res* **9**: 1143–1155.

Murata K, Yasumasu S, Lee YM, Hedrick JL. 2000. Fish egg lectins; Important factors for a polyspermy block during fertilization. *Mol Biol Cell* **11**: A405–A405 2103 Suppl.

Neto ED, Correa RG, Verjovski-Almeida S, *et al*. 2000. Shotgun sequencing of the human transcriptome with ORF expressed sequence tags. *Proc Natl Acad Sci USA* **97**: 3491–3496.

Neto ED, Harrop R, CorreaOliveira R, *et al*. 1997. Minilibraries constructed from cDNA generated by arbitrarily primed RT-PCR: an alternative to normalized libraries for the generation of ESTs from nanogram quantities of mRNA. *Gene* **186**: 135–142.

Onichtchouk D, Aduroja K, Belting HG, Gnugge L, Driever W. 2003. Transgene driving GFP expression from the promoter of the zona pellucida gene zpc is expressed in oocytes and provides an early marker for gonad differentiation in zebrafish. *Dev Dyn* **228**: 393–404.

Ozeki Y, Yokota Y, Kato KH, Titani K, Matsui T. 1995. Developmental expression of D-galactoside-binding lectin in sea urchin *(Anthocidaris crassispina)* eggs. *Exp Cell Res* **216**: 318–324.

Pijnacker LP, Ferwerda MA. 1995. Zebrafish chromosome banding. *Genome* **38**: 1052–1055.

Reinke V, Smith HE, Nance J, *et al*. 2000. A global profile of germline gene expression in *C. elegans*. *Mol Cell* **6**: 605–616.

Rockett JC, Luft JC, Garges JB, *et al*. 2001. Development of a 950-gene DNA array for examining gene expression patterns in mouse testis. *Genome Biol* **2**: 14.1–14.9.

Schummer M, Ng VLV, Baumgarner RE, *et al*. 1999. Comparative hybridization of an array of 21 500 ovarian cDNAs for the discovery of genes overexpressed in ovarian carcinomas. *Gene* **238**: 375–385.

Sigrist CJ, Cerutti L, Hulo N, *et al*. 2002. PROSITE: a documented database using patterns and profiles as motif descriptors. *Brief Bioinform* **3**: 265–274.

Sola L, Gornung E. 2001. Classical and molecular cytogenetics of the zebrafish, *Danio rerio* (Cyprinidae, Cypriniformes): an overview. *Genetica* **111**: 397–412.

Spargo SC, Hope RM. 2003. Evolution and nomenclature of the zona pellucida gene family. *Biol Reprod* **68**: 358–362.

Takahashi H. 1977. Juvenile hermaphroditism in the zebrafish, *Brachydanio rerio. Bull Fac Fish Hokkaido Univ* **28**: 57–65.

Tateno H, Ogawa T, Muramoto K, *et al*. 2001. A novel rhamnose-binding lectin family from eggs of steelhead trout (*Oncorhynchus mykiss*) with different structures and tissue distribution. *Biosci Biotechnol Biochem* **65**: 1328–1338.

Tateno H, Ogawa T, Muramoto K, Kamiya H, Saneyoshi M. 2002. Distribution and molecular evolution of rhamnose-binding lectins in *Salmonidae*: isolation and characterization of two lectins from white-spotted charr (*Salvelinus leucomaenis*) eggs. *Biosci Biotechnol Biochem* **66**: 1356–1365.

Tateno H, Saneyoshi A, Ogawa T, *et al*. 1998. Isolation and characterization of rhamnose-binding lectins from eggs of steelhead trout (*Oncorhynchus mykiss*) homologous to low density lipoprotein receptor superfamily. *J Biol Chem* **273**: 19 190–19 197.

Thompson JD, Higgins DG, Gibson TJ. 1994. Clustal-W — improving the sensitivity of progressive multiple sequence alignment through sequence weighting, position- specific gap penalties and weight matrix choice. *Nucleic Acids Res* **22**: 4673–4680.

Ton C, Hwang DM, Dempsey AA, *et al*. 2000. Identification, characterization, and mapping of expressed sequence tags from an embryonic zebrafish heart cDNA library. *Genome Res* **10**: 1915–1927.

Copyright © 2004 John Wiley & Sons, Ltd. 

Ton C, Stamatiou D, Dzau DJ, Liew C-C. 2002. Construction of a zebrafish microarray: gene expression profiling of the zebrafish during development. *Biochem Biophys Res Comm* **296**: 1134–1142.

Uchida D, Yamashita M, Kitano T, Iguchi T. 2002. Oocyte apoptosis during the transition from ovary-like tissue to testes during sex differentiation of juvenile zebrafish. *J Exp Biol* **205**: 711–718.

Vos P, Hogers R, Bleeker M, *et al.* 1995. AFLP: a new technique for DNA fingerprinting. *Nucleic Acids Res* **23**: 4407–4414.

Wakimoto BT. 2000. Doubling the rewards: testis ESTs for *Drosophila* gene discovery and spermatogenesis expression profile analysis. *Genome Res* **10**: 1841–1842.

Wassarman P, Chen J, Cohen N, *et al.* 1999. Structure and function of the mammalian egg zona pellucida. *J Exp Zool* **285**: 251–258.

Woods IG, Kelly PD, Chu F, *et al.* 2000. A comparative map of the zebrafish genome. *Genome Res* **10**: 1903–1914.

Wu XM, Viveiros MM, Eppig JJ, *et al.* 2003. Zygote arrest 1 (Zar1) is a novel maternal-effect gene critical for the oocyte-to-embryo transition. *Nature Genet* **33**: 187–191.

Yasumasu S, Wardrip NJ, Zenner BD, *et al.* 2000. Fertilisation in fish: a cortical alveolar lectin and its potential role in the block to polyspermy. *Zygote* **8**: S66–S66.

Zeng S, Gong Z. 2002. Expressed sequence tag analysis of expression profiles of zebrafish testis and ovary. *Gene* **294**: 45–53.

Copyright © 2004 John Wiley & Sons, Ltd. 
